# Focusing on the role of secretin/adhesion (Class B) G protein-coupled receptors in placental development and preeclampsia

**DOI:** 10.3389/fcell.2022.959239

**Published:** 2022-09-14

**Authors:** Aiqi Yin, Xiaonian Guan, Jian V. Zhang, Jianmin Niu

**Affiliations:** ^1^ Department of Obstetrics, Shenzhen Maternity and Child Healthcare Hospital, The First School of Clinical Medicine, Southern Medical University, Shenzhen, China; ^2^ Center for Energy Metabolism and Reproduction, Shenzhen Institute of Advanced Technology, Chinese Academy of Sciences, Shenzhen, China; ^3^ Shenzhen Institute of Advanced Technology, Chinese Academy of Sciences, Shenzhen, China; ^4^ Shenzhen Key Laboratory of Metabolic Health, Shenzhen, China

**Keywords:** preeclampsia (PE), pathogenesis, placenta, secretin GPCRs, adhesion GPCRs

## Abstract

Preeclampsia, a clinical syndrome mainly characterized by hypertension and proteinuria, with a worldwide incidence of 3–8% and high maternal mortality, is a risk factor highly associated with maternal and offspring cardiovascular disease. However, the etiology and pathogenesis of preeclampsia are complicated and have not been fully elucidated. Obesity, immunological diseases and endocrine metabolic diseases are high-risk factors for the development of preeclampsia. Effective methods to treat preeclampsia are lacking, and termination of pregnancy remains the only curative treatment for preeclampsia. The pathogenesis of preeclampsia include poor placentation, uteroplacental malperfusion, oxidative stress, endoplasmic reticulum stress, dysregulated immune tolerance, vascular inflammation and endothelial cell dysfunction. The notion that placenta is the core factor in the pathogenesis of preeclampsia is still prevailing. G protein-coupled receptors, the largest family of membrane proteins in eukaryotes and the largest drug target family to date, exhibit diversity in structure and function. Among them, the secretin/adhesion (Class B) G protein-coupled receptors are essential drug targets for human diseases, such as endocrine diseases and cardiometabolic diseases. Given the great value of the secretin/adhesion (Class B) G protein-coupled receptors in the regulation of cardiovascular system function and the drug target exploration, we summarize the role of these receptors in placental development and preeclampsia, and outlined the relevant pathological mechanisms, thereby providing potential drug targets for preeclampsia treatment.

## Introduction

Preeclampsia (PE) is a multisystem pregnancy disorder characterized by new-onset hypertension after 20 weeks of gestation and affects the functions of multiple organ (Brown et al., 2018; Chappell et al., 2021). PE influences around 3–8% of the pregnant women and remains a key cause of maternal mortality, bringing about at least 42,000 maternal deaths each year (Abalos et al., 2013; Say et al., 2014). PE seriously threatens maternal and fetal life safety, leading to many severe complications such as acute renal failure, intracranial hemorrage, fetal growth restriction (FGR), abnormal fetal heart development and still birth (Adu-Bonsaffoh et al., 2013; Brown et al., 2018; Hutcheon et al., 2011). Furthermore, PE can bring substantial long-term cardiovascular and endocrine metabolic risks both to the mother and the child (Bellamy et al., 2007; Kajantie et al., 2009; Mongraw-Chaffin et al., 2010; Tuovinen et al., 2012). The strong evidence has demonstrated that aspirin can prevent the development of PE (Duley et al., 2019; Jin, 2021). However, once PE occurs, the existing treatments, such as antispasmodic and antihypertensive agents, cannot prevent the progression of the disease. The termination pf pregnancy is the only effective treatment for PE, which may leads to the delivery of premature fetus or low birth weight fetus, with high healthcare costs (Chappell et al., 2021; Liu et al., 2009). Thus, it is necessary to explore new treatments for PE to reduce the risk and safely prolong pregnancy.

The pathophysiological mechanisms of PE have been studied for a long time. The placenta is the key factor responsible for the development of PE. All maternal complications share a common pathophysiological feature focusing on placental abnormalities (Burton et al., 2019). The pathogenesis of PE contains poor placentation, uteroplacental malperfusion and endothelial cell dysfunction (Brennan et al., 2014; Redman and Staff, 2015). However, due to the heterogeneity of PE and the diversity of its clinical manifestations, the immunological, genetic and environmental mechanisms of PE are still not fully understood, and there is no great breakthrough regarding the treatment for PE. Some research scholars advocate that PE should be reclassified using placenta-derived markers or new phenotypic combinations, which may assist in identifying high-risk patients, monitoring disease progression, and providing effective clinical interventions (Ferrazzi et al., 2018; Powers et al., 2012).

G protein-coupled receptors (GPCRs), the largest family of membrane proteins in eukaryotes, are involved in the regulation of almost all life processes and functions. GPCRs are the key factors for the occurrence and development of major diseases, including cardiometabolic diseases (Dorsam and Gutkind, 2007; Lappano and Maggiolini, 2011; Wang et al., 2018). Exploratory studies have revealed the essential functions of GPCRs in placental development and provided a sufficient theoretical basis that they can be used as potential targets for PE (Conrad, 2016; McGuane and Conrad, 2012; Quitterer and AbdAlla, 2021). Based on the classification by structure and phylogeny analysis of GPCRs, Class B GPCRs, which are structurally characterized by large extracellular regions, contain the following two families, secretin GPCRs and adhesion GPCRs. Secretin GPCRs are polypeptide hormone receptors that can mediate diverse physiological activities. Adhesion GPCRs are indispensable for human development, and their mutations are involved in all major tissues diseases. This review summarizes the pathophysiological mechanisms of PE and the biological role of secretin/adhesion (Class B) GPCRs in placental development and PE, aiming to find potential markers for the reclassification and treatment of PE.

## Pathophysiology of PE: the two-stage placental model

**FIGURE 1:**
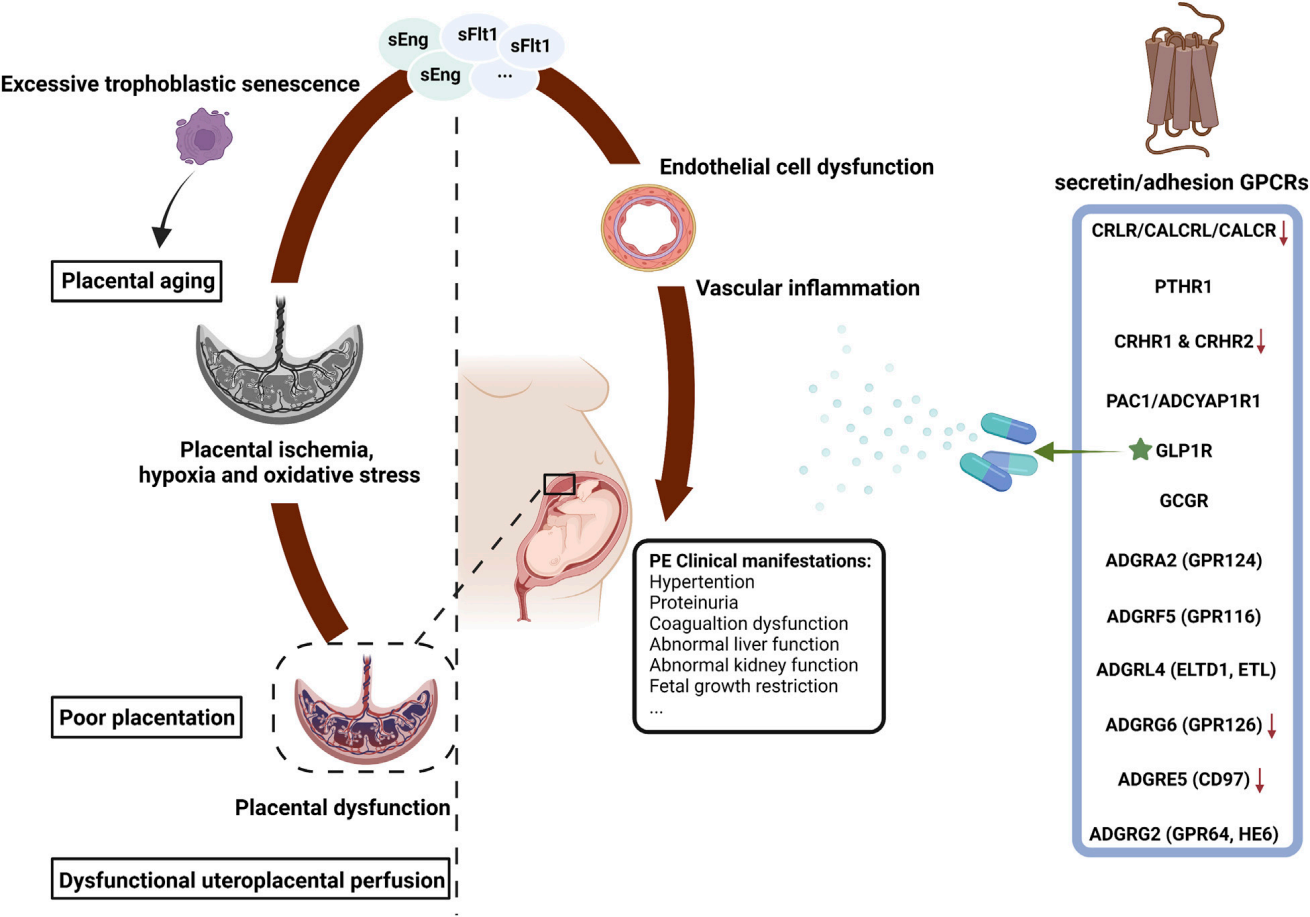
The secretin/adhesion GPCRs and the pathophysiology of preeclampsia. PE, preeclampsia; GPCRs, G protein-coupled receptors; sFlt-1, soluble fms-like tyrosine kinase 1; sEng, soluble endoglin; FGR, fetal growth restriction; ★, commercial drug; ↓, expressed at low levels in PE. The figure was created with BioRender.com.

The clinical symptoms of PE are immediately relieved once the placenta is delivered, suggesting that the placenta is crucial for the pathogenesis of PE. The two-stage placental model theory proposed by professor Redman is the most acceptable explanation for the pathogenesis of PE (Redman, 1991). In the first stage (preclinical and placental period), insufficient trophoblast infiltration causes the incomplete remodeling of the uterine spiral arteries, resulting in poor placentation and placental dysfunction. In the second stage (clinical and maternal disease period), placental ischemia, hypoxia and oxidative stress lead to the release of numerous inflammatory factors into the circulation, including soluble fms-like tyrosine kinase-1 (sFlt-1), soluble endoglin (sEng), trophoblast debris and reactive oxygen species (Levine et al., 2004; Maynard et al., 2003). These factors cause systemic vascular inflammation and extensive maternal endothelial cell dysfunction (Redman et al., 1999) and provoke diverse clinical manifestations such as maternal hypertension, proteinuria and FGR (Powe et al., 2011). This two-stage model is established on the assumption that poor placentation, predominantly leading to FGR, occurs in almost all PE cases (Avagliano et al., 2011). However, some of the late-onset PE patients with full delivery show unlimited neonatal growth, which suggests that poor placentation does not happens in all PE placenta (Xiong et al., 2002). The second placental cause of PE is uteroplacental malperfusion. The placenta capacity over the capacity of the uterus could compress the terminal villi and hinder intervillous perfusion, resulting in syncytiotrophoblast hypoxia and the subsequent placental dysfunction (Devisme et al., 2013). Increased syncytiotrophoblast apoptosis in the human full-term placenta may cause PE as well (Ishihara et al., 2002). Studies have shown that the angiogenesis-related factors secreted by syncytiotrophoblasts are expected to be circulating biomarkers for the diagnosis and prediction of PE (Droge et al., 2021). Excessive trophoblastic senescence increases placental cell stress, which may be a potential pathogenic factor for PE (Zaki et al., 2003; Zhang et al., 2021). Overall, syncytiotrophoblast stress is a common end point of both early-onset and late-onset PE pathways and is affected by maternal genetic, epigenetic and environmental factors. Syncytiotrophoblast stress signaling in the maternal circulation may be the most specific biomarker of PE (Redman et al., 2022). The two-stage model summarizes the pathophysiological mechanisms of PE into two stages (placental dysfunction and clinical manifestations) and three ways (poor placentation, dysfunctional uteroplacental perfusion and placental aging) (Figure 1). In addition, it incorporates a range of pathophysiological mechanisms, including dysregulated immune tolerance, vascular inflammation, endoplasmic reticulum stress and oxidative stress (Sheppard and Bonnar, 1976). Maternal and pregnancy risk factors, such as primiparity, obesity and chronic prepregnancy disorders, are also considered (Alnaes-Katjavivi et al., 2016; Egeland et al., 2016; Skjaerven et al., 2002; Tannetta et al., 2015).

## Role of GPCRs in PE

GPCRs, a superfamily of seven-transmembrane receptors, more than 800 of which are encoded in the human genome, constitute the largest family of cell surface receptors in mammalian cells. GPCRs were initially divided into A–F systems according to the structural similarity of their receptor size, ligand interaction points and phylogeny. Their prototype members are as follows: Class A (rhodopsin receptors), Class B (secretin/adhesion receptors), Class C (metabotropic glutamate receptors), Class D (fungal mating pheromone receptors), Class E (cyclic AMP receptors) and Class F (frizzled/smoothened receptors) (Attwood and Findlay, 1994; Bockaert and Pin, 1999). Fredriksson et al. provided a GRAFS classification approach based on an overall phylogenetic analysis of human tracks that consists of the following five families: rhodopsin (Class A), secretin/adhesion (Class B), glutamate (Class C), frizzled (Class F) and taste 2 (Class T) (Fredriksson et al., 2003). GPCRs can recognize various different ligands or stimuli, such as hormones, neurotransmitters and light, to regulate key physiological processes. Abnormal GPCR signaling can lead to various diseases, such as diabetes (Riddy et al., 2018; Wu et al., 2021), cardiovascular diseases (Wang et al., 2018) and cancers (Dorsam and Gutkind, 2007; Lappano and Maggiolini, 2011). More than 40% of commercial drugs exert their efficacy through GPCRs. Most drugs target rhodopsin GPCRs because they have various ligands, but other GPCRs also possess distinct therapeutic potential, such as more than 34 drugs targeting secretin GPCRs and 21 drugs targeting glutamate GPCRs (Hutchings, 2020). Furthermore, research on drugs targeting GPCRs are still increasing (Zhou and Wild, 2019).

The GPCRs-targeted drugs such as GLP-1, which belongs to secretin GPCRs, have been used for the treament of cardiovascular diseases and endocrine diseases. The GPCR-mediated regulation of vascular tone and circulating blood volume plays crucial roles in the maintenance of blood pressure homeostasis. PE is inextricably linked to endocrine and cardiovascular diseases. Recently, it’s found GPCRs potentially contribute to maternal physiological adaptation to pregnancy and placental development. Several GPCRs such as calcitonin receptor-like receptor (CRLR) and angiotensin AT1/2 are potential therapeutic targets for PE. They can modulate systemic and/or uteroplacental vasodilation to alleviate hypertension in PE (Dong et al., 2006). Secretin and adhesion GPCRs, which belong to the class B GPCR family, present great potential in clinical use. Many studies are committed to investigate the fundamental role of Class B (secretin/adhesion) GPCRs in PE, so it is necessary to summarize the current research progress. A comprehensive summary of the researches in relation to Class B (secretin/adhesion) GPCRs in the field of placental development and PE is provided in the following part of this review (Table 1).

**TABLE 1:** The secretin/adhesion (class B) GPCRs in placental development and PE.

Secretin (Class B) GPCRs	Species	GPCRs in placental development and PE	References
*CRLR/CALCRL/CALCR*	Human	Lowly expressed at the uterus and umbilical artery in pregnancy-induced hypertension	Makino et al. (2001)
	Human	Expressed at the vascular endothelial cells of placental chorionic villi in the first trimester	Tsatsaris et al. (2002)
	Mouse	Expressed in placenta and involved in blood flow	Dong et al. (2003)
	Human	Regulates human fetoplacental vascular tone	Dong et al. (2004)
	Human	Expressed in human trophoblast cell line JAr and HTR-8/SVneo	Zhang et al. (2005)
	Human	Decreasingly expressed in preeclamptic placenta	Dong et al. (2005)
		Affects vasodilation in PE	
	Mouse	Leads to hydrops fetalis, cardiovascular defects and embryonic lethality in CALCRL KO mouse	Dackor et al. (2006)
	Mouse	Shows a spatiotemporal pattern in rat female reproductive system	Li et al. (2010)
	Human	Results in autosomal recessive, hydrops fetalis and lymphatic dysplasia with CALCRL mutation	Mackie et al. (2018)
*PTHR1*	Human	Inhibits proliferation of JEG-3 cell line	Hellman et al. (1993)
	Mouse	Decreasingly expressed via CALCRL-associated RAMP2 regulation	Kadmiel et al. (2017)
	Human	Highly expressed in extravillous cytotrophoblast and decidua	Sirico et al. (2021)
		Expressed differently depending on maternal hyperglycemia type	
*CRHR1 & CRHR2*	Human	Expressed in syncytiotrophoblast cells and amniotic epithelium	Karteris et al. (1998)
	Human	Decreased expression of CRHR1 in PE and IUGR	Karteris et al. (2003)
		Influences vascular resistance	
	Human	Located in cultured human chorion trophoblast cells	Gao et al. (2007)
		Mediates expression of PGDH	Gao et al. (2008)
	Human	Expressed in placental trophoblasts	Gao et al. (2012)
		Regulates prostaglandin production	
	Human	Regulates estradiol and progesterone production in cultured human trophoblasts	Gao et al. (2012)
	Human	Regulates glucose transporters in cultured human placental trophoblasts	Gao et al. (2012)
	Human	Increasingly expressed via exogenous CRH stimulation in BeWo cells	Chen et al. (2013)
*GCGR*	Mouse	Causes hypoglycemia, hyperglucagonemia and fetoplacental defects in GCGR KO mouse	Ouhilal et al. (2012)
*GLP1R*	Mouse	Attenuates placental ischemia	Younes et al. (2020)
*PAC1/ADCYAP1R1*	Human/mouse	Expressed in human and rat placenta	Scaldaferri et al. (2000)
	Mouse	As binding sites for PACAP in human tissue	Koh et al. (2003)
		Expressed in decidual cells, chorionic vessels and stromal cells	
		Dynamically expressed during gestation	
	Human	Expressed in stroma cells with spatiotemporal characteristics	Koh et al. (2005)
	Human	Regulates MAPK signaling pathways in cytotrophoblast cells	Reglodi et al. (2008)
**Adhesion (Class B) GPCRs**	**Species**	**GPCRs in placental development and preeclampsia**	**References**
*ADGRA2* (*GPR124*)	Mouse	Causes embryonic lethality, CNS-specific angiogenesis arrest and hemorrhage in GPR124 KO mouse	Kuhnert et al. (2010)
	Mouse	Results in embryonic lethality in GPR124 global KO mouse and GPR124 conditional (endothelial-specific) KO mouse	Cullen et al. (2011)
	Human	Lowly expressed in early-onset PE with comparison of late-onset PE	Liang et al. (2016)
	Mouse	Leads to embryonic lethality in GPR124 KO mouse	Chang et al. (2017)
*ADGRF5* (*GPR116*)*, ADGRL4* (*ADGRG6 (GPR126)*)	Mouse	Causes vascular remodeling defects and postnatal kidney failure	Lu et al. (2017)
	Mouse	Possesses strict expression pattern	Moriguchi et al. (2004)
	Mouse	Required for embryonic development	Waller-Evans et al. (2010)
	Human	Related to placental angiogenesis in IUGR	Majewska et al. (2019)
	Mouse/zebrafish	Leads to embryonic lethality in GPR126 KO mouse	Torregrosa-Carrion et al. (2021)
		Expressed in trophoblast giant cells	
		Regulates trophoblast invasion and spiral artery remodeling	Bogias et al. (2022)
		Affects the expression of PE markers in GPR126 KO placenta	
	Human	Related to hypoxia at early pregnancy	
*ADGRE5* (*CD97*)	Human	Lowly expressed in PE placenta	Shen et al. (2020)
		Promotes trophoblast invasion through PI3K/Akt/mTOR pathway	
*ADGRG2* (*GPR64, HE6*)	Mouse	Expressed at epithelial and stromal cells in the uterus	Yoo et al. (2017)
		Reduces decidualization in GPR64 KO mouse	

**Note**: GPCRs, G protein-coupled receptors; PE, preeclampsia; KO, Knockout; RAMPs, Receptor activity-modifying protein 2; IUGR, intrauterine growth restriction; PGDH, 15-hydroxy prostaglandin dehydrogenase; CRH, Corticotropin-releasing hormone; PACAP, Pituitary adenylate cyclase-activating polypeptide; CNS, central nervous system.

### Secretin (Class B) GPCRs

The secretin family, a small part of GPCRs containing 15 members, has large extracellular domains that can bind to hormone and mainly regulates metabolism (Lagerstrom and Schioth, 2008).

Calcitonin receptor-like receptor (CRLR) is required for embryonic development (Chang and Hsu, 2013). Deficiency of CRLR causes extreme hydrops fetalis and embryonic death (Dackor et al., 2006; Mackie et al., 2018). CRLR is widely expressed in vascular endothelial cells of placental chorionic villi at the first trimester, human choriocarcinoma JAr cells and trophoblast HTR-8/svneo cells (Tsatsaris et al., 2002). It shows a spatiotemporal pattern in the female reproductive system of pregnant rats (Li et al., 2010). An analysis of mouse placenta obtained on E18 of pregnancy suggests that CRLR is predominantly expressed in trophoblast, syncytiotrophoblast and trophoblast giant cells (Chang and Hsu, 2013; Dong et al., 2003; Zhang et al., 2005). CRLR regulates trophoblast proliferation and differentiation in the implantation process (Tsatsaris et al., 2002). Existing research demonstrates that CRLR is decreasingly expressed at the uterus and umbilical artery tissues in pregnancy-induced hypertension patients (Makino et al., 2001) and at PE placenta (Dong et al., 2005). It also associates with PE in vascular remodeling (Chang and Hsu, 2013). The impairment of CRLR associated with calcitonin gene-related peptide (CGRP)-dependent vasodilation in PE (Dong et al., 2005) suggest their role in the control of human fetoplacental vascular tone. CGPR-mediated vascular dilation involves the activation of KATP channels, cAMP and nitric oxide pathway (Dong et al., 2004). The loss of CRLR associated receptor activity-modifying proteins (RAMPs) reduces the expression of parathyroid hormone 1 receptor (PTHR1) (Kadmiel et al., 2017). PTHR1 expresses differently depending on maternal hyperglycemia type. It’s highly expressed in the extravillous cytotrophoblasts and decidua tissues, regulating the human trophoblast-derived JEG-3 cell proliferation (Hellman et al., 1993), and committing to adverse pregnancy outcomes (Sirico et al., 2021).

Corticotropin releasing hormone (CRH) promotes embryo implantation (Makrigiannakis et al., 2001). It also control trophoblast invasion by downregulating the synthesis of carcinoembryonic antigen-related cell adhesion molecule 1 (CEACAM1) in extravillous trophoblast (EVT) cells (Bamberger et al., 2006). Abnormalities in trophoblast invasion may lead to abnormal placentation. The treatment of BeWo cells with exogenous CRH results in the elevation of cellular corticotropin releasing hormone receptor 1 (CRHR1) levels, which are significantly reduced in PE and intrauterine growth restriction (IUGR) (Chen et al., 2013; Karteris et al., 2003). CRHR1 and CRHR2 are expressed in placental trophoblasts, and they regulate estradiol and progesterone production as well as glucose transporters through distinct signaling pathways. They exert differential effects on 15 hydroxy prostaglandin dehydrogenase (PGDH) expression (Gao et al., 2007; Gao et al., 2008; Gao et al., 2012a; Gao et al., 2012b; Karteris et al., 1998). The deficiency of CRHR1 in uterine at early pregnancy implicates the pathogenesis of recurrent miscarriage, placenta accreta and PE (Kalantaridou et al., 2007).

The absence of the glucagon receptor (GCGR) gene during pregnancy leads to abnormal placentation and poor fetal growth, increasing occurrence rate of fetal and early postnatal death. The placenta affected by GCGR are characterized by extensive mineralization, fibrinoid necrosis, narrowing of the vascular channels and thickened interstitium associated with trophoblast hyperplasia. In addition, the lack of GCGR downregulates genes that control growth, vascularization and oxidative stress (Charron and Vuguin, 2015; Ouhilal et al., 2012). Regarding glucagon-like peptide receptors (GLPRs, including GLP1R and GLP2R), it’s found that the GLP1R agonist liraglutide can increase nitric oxide production and decrease blood pressures. They function partially through activating nitric oxide synthase (NOS) and thus serve as a potential therapeutic option for PE (Younes et al., 2020).

Pituitary adenylate cyclase activating polypeptide receptor 1 (PAC1), expressed in both human and mouse placenta, has spatiotemporal expression characteristics in decidual cells, chorionic vessels and stromal cells (Koh et al., 2003; Koh et al., 2005; Scaldaferri et al., 2000). Its antagonist PACAP6-38 can activate MAPK signaling in human cytotrophoblasts, which suggests the possible role in gestational maintenance and fetal growth (Reglodi et al., 2008). Drugs designed on the basis of secretin GPCR have been developed and applied for clinical use, especially in the treatment of metabolic diseases, such as diabetes. Given diabetes and obesity are risk factors for PE, the prospect of secretin GPCRs in therapy of PE could be speculated.

### Adhesion (Class B) GPCRs

The adhesion family of GPCRs, a large branch with 33 members, is the second largest family of GPCRs separated from secretin GPCRs. The International Union of Basic and Clinical Pharmacology (IUPHAR) rename adhesion GPCRs as ADGRs followed a letter and a number to denote their subfamily and subtype, respectively (Hamann et al., 2015). Adhesion GPCRs are paid close attention due to its specific biological function and structure. Most adhesion GPCRs have long diverse N termini, and their N termini are rich in functional domains that can be found in other proteins, such as cadherins, lectins and immunoglobulins. It’s shown that the number and structure of these domains are essential for the specificity of receptor ligand binding interactions (Bjarnadottir et al., 2004; Purcell and Hall, 2018).

Several adhesion GPCRs participate in angiogenesis, a process that implicates in gestational physiology, placental development and the occurrence of PE (Masiero et al., 2013; Stehlik et al., 2004; Vallon and Essler, 2006; Wang et al., 2005). ADGRA2 (GPR124) deficiency leads to embryonic lethality due to the central nervous system (CNS)- specific angiogenesis and hemorrhage (Anderson et al., 2011; Chang et al., 2017; Cullen et al., 2011; Kuhnert et al., 2010). The loss of ADGRF5 (GPR116) and ADGRL4 (ELTD1, ETL) result in vascular remodeling defects (Lu et al., 2017). Both ADGRA2 (GPR124) and ADGRG6 (GPR126) are required for embryonic development (Chang et al., 2017; Waller-Evans et al., 2010). ADGRG6 (GPR126) has a strictly regulated expression pattern in mouse development (Moriguchi et al., 2004). A differential expression of ADGRG6 (GPR126) is found in IUGR placenta, which is correlated with placental angiogenesis (Majewska et al., 2019) and hypoxia in early pregnancy (Bogias et al., 2022). ADGRG6 (GPR126) mutant placenta shows a decreased expression of proteases associated with trophoblast invasion and maternal uterine vascular remodeling, leading to IUGR, PE and early miscarriage. Hence, ADGRG6 (GPR126) is essential in the trophoblast lineage for the promotion of spiral artery remodeling during placental development (Torregrosa-Carrion et al., 2021). Our research group previously analyzes the gene expression profiles at placentas in early-onset PE and late-onset PE. ADGRA2 (GPR124) is downregulated in early-onset PE and involve in cell surface receptor-related signaling (Liang et al., 2016). The specific role and mechanism of ADGRA2 (GPR124) in placental development and PE are still under investigation. ADGRE5 (CD97) is downregulated in PE placenta and promotes trophoblast invasion by targeting FOXC2 through PI3K/Akt/mTOR signaling pathway (Shen et al., 2020). ADGRG2 (GPR64, HE6) plays a crucial role in the decidualization of endometrial stromal cells (Yoo et al., 2017). Adhesion GPCRs are considered as suitable targets for therapy, but the ligands of most members have not been found. And there are no associated drugs are currently approved by the FDA.

## Conclusion and perspectives

In summary, the Class B (secretin/adhesion) GPCRs play crucial roles in placental development and PE, suggesting that the class B (secretin/adhesion) GPCRs could serve as therapeutic targets in PE. However, most of the studies mainly focus on their expression level and associated phenotype, whereas the molecular mechanisms still need to be paid much more attention. Deep comprehension on the mechanisms is required to provide solid rationale for the application of Class B (secretin/adhesion) GPCR-targeted drugs into PE.
